# Atrial septal aneurysm associated with additional cardiovascular comorbidities in two middle age female patients with ECG signs of right bundle branch block: two case reports

**DOI:** 10.1186/1757-1626-1-51

**Published:** 2008-07-19

**Authors:** Aurora Bakalli, Lulzim Kamberi, Ejup Pllana, Afrim Gashi

**Affiliations:** 1 University Clinical Center of Kosova-Internal Clinic, Department of Cardiology, Prishtine, Kosove; 2 University Clinical Center of Kosova-Internal Clinic, Department of Rheumatology, Prishtine, Kosove

## Abstract

**Introduction:**

Atrial septal aneurysm (ASA) is often associated with other atrial septal abnormalities, particularly with atrial septal defect type *ostium secundum *or patent foramen ovale. ECG signs of incomplete or complete right bundle branch block are known to be associated with atrial septal defects, however such correlation with other atrial septal abnormalities is not documented.

**Case presentations:**

We report here two cases of middle age female patients that presented with dyspnea on physical effort, right bundle branch block (RBBB) on ECG and ASA combined with other cardiac disorders. Transesophageal echocardiography revealed additional information to the ones obtained by surface echocardiography, in both cases.

**Conclusion:**

ASA associated with RBBB on ECG may serve as a hint for the presence of additional cardiac abnormalities, thus rousing the demand for a detailed cardiac investigation.

## Introduction

Initially it was thought that Atrial Septal Aneurysm (ASA) is a rare congenital anomaly, but with the advancement of two-dimensional echocardiography and lately with broader use of transesophageal echocardiography (TEE), detection of this abnormality has become easier and more frequent. Studies using TEE have shown a prevalence of 2% to 10% for ASA [1,2]. ASA is frequently associated with other atrial septal defects, in particular Atrial Septal Defect (ASD) type *ostium secundum *and Patent Foramen Ovale (PFO) [3]. Another type of cardiac abnormality often associated with ASA is Mitral Valve Prolapse (MVP) [4].

Right bundle branch block (RBBB) is known to be associated with atrial septal defects, on the other hand there are contradictions of its correlation with PFO [5]. Association between ASA and atrial tachyarrhythmias has been suggested [6], nevertheless there are no studies, to our awareness, that correlate complete or incomplete RBBB with ASA.

We describe here two cases of female patients complaining of dyspnea on physical effort, presenting with signs of RBBB on ECG and presence of ASA combined with other cardiovascular disorders.

## Case presentations

### Case 1

A 45 year old white female patient presented in the cardiology outpatient clinic with complaints of breathlessness on higher levels of physical exertion, which started one month earlier. She was a housewife and a mother of three children. Patient did not smoke or use alcohol. She didn't have a history of other diseases, denied usage of any medications and was not aware of the presence of heart diseases among her family members.

Patient's height was 158 cm and she weighed 52 kg. Her vital signs were as following: blood pressure 110/70 mmHg; pulse rate 70 beats/min.; respiration rate 16/min.; body temperature 36.5°C. Heart auscultation revealed regular heart rhythm, clear sounds and systolic click on the apex. On lung auscultation there was normal breathing, without rales or wheezes. Other systems had no remarks on physical examination.

Laboratory findings were within normal reference range.

ECG revealed sinus rhythm, heart rate of 65/min., slight right axis deviation and RBBB (Figure 3).

**Figure 1:**
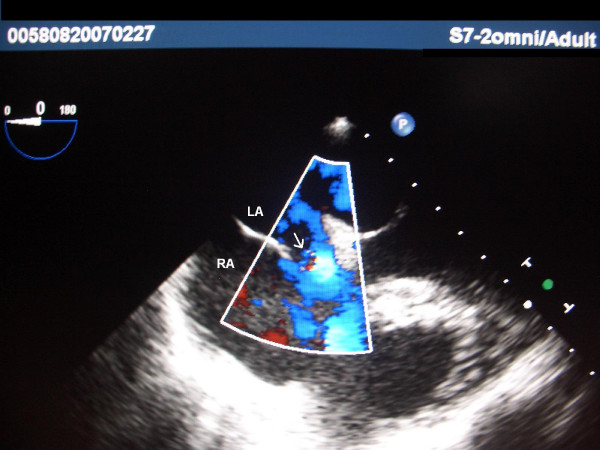
**TEE image 1 of the first patient**. Color Doppler imaging depicts the interatrial shunting (arrow). LA – Left Atrium; RA – Right Atrium.

**Figure 2:**
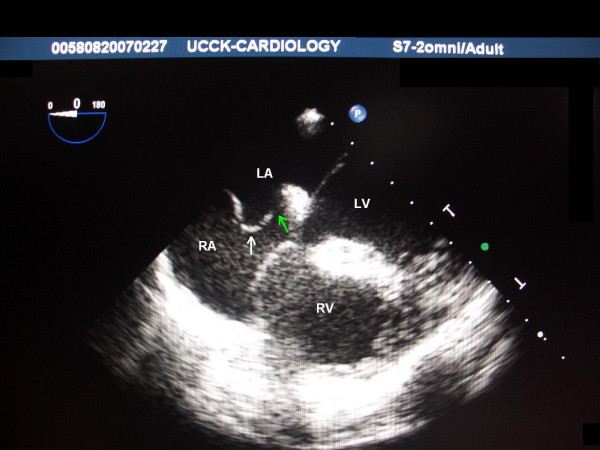
**TEE image 2 of the first patient**. TEE image demonstrating an atrial septal aneurysm protruding into the right atrium (white arrow) and a gap between the septa consistent with patent foramen ovale (green arrow). *Septum primum *appears very thin in this image. LA – Left Atrium; RA – Right Atrium; LV – Left Ventricle; RV – Right Ventricle.

Based on these findings, patient was appointed for echocardiography. Echo findings revealed mitral valve prolapse, aneurysm of atrial septum, enlarged right ventricle and a mild elevation of pulmonary artery systolic pressure (PASP). Other data obtained by echocardiography are presented in Table 1. Due to the presence of ASA type 1R, we decided to further investigate the atrial septum and to perform a TEE. Apart from confirming the abnormalities detected by transthoracic echocardiography, an interatrial shunt was exposed by TEE (Figure 1). This shunting was due to the presence of PFO that was visualized intermittently as a tunnel-like gap between the two septa, which in its maximal opening had a distance of 4.5 mm (Figure 2).

Low doses of Hydrochlorothiazide, due to mild right heart failure, and Aspirin, for prevention of thromboembolic events, were given. One year later the patient was feeling better and she was left on Aspirin therapy, only.

### Case 2

A 57 year old Caucasian woman presented complaining of dyspnea on low level physical effort, fatigue, intermittent dry cough, and swelling of the legs. Symptoms had started years ago but were intensified during the last three months. This was her first visit to a cardiology clinic. Patient was a housewife and did not have children. The patient denied tobacco use, alcohol use or usage of medications. She had no family history of cardiac illnesses.

Patient's height was 162 cm and she weighed 60 kg. Her vital signs were: blood pressure 120/70 mmHg; pulse rate 135 beats/min.; respiration rate 18/min.; body temperature 37°C.

On physical examination of the head and neck, distension of the jugular veins was noted. On auscultation of the chest, her lung sounds were clear, without rales. Her heart sounds were irregular, increased pulmonic component of the second heart sound was present, systolic murmur 4/6 at the left lower sternal border, systolic murmur 3/6 at the apex, diastolic murmur 2/6 at the second right intercostal space close to the sternum were heard. Pretibial edema 2+ was present bilaterally. No significant findings were obtained from the examination of other systems.

Laboratory results were within normal limits.

ECG showed atrial fibrillation, ventricular response of 135/min., right axis deviation, RBBB. Chest X-ray revealed moderate cardiomegaly of the right side.

Echocardiography demonstrated dilation of the right heart chambers, ASA type 1R, a 16 mm ASD type *ostium primum*, mitral regurgitation 2+, tricuspid regurgitation 3+, elevated PASP of 60 mmHg and aortic regurgitation 1+. Other echocardiography parameters are presented in Table 1. TEE was also performed and in addition to the above mentioned disorders (Figure 4), a smaller size ASD type *ostium secundum *was detected (Figures 5 and 6).

**Figure 3:**
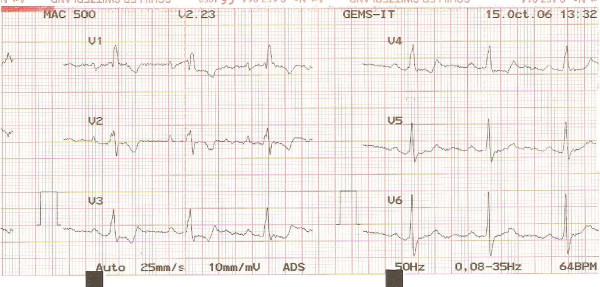
**ECG of the first patient**. Precordial ECG leads showing signs of RBBB.

**Figure 4:**
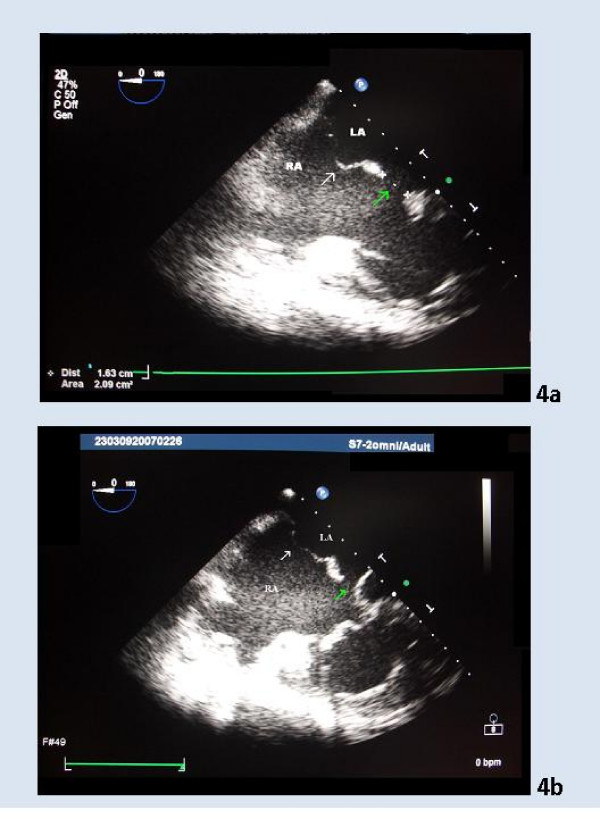
**4a and 4b. Two TEE images of the second patient**. TEE image of the second patient demonstrating an atrial septal aneurysm type 1R (white arrow) and a 16.3 mm *ostium primum *septal defect (green arrow). LA – Left Atrium; RA – Right Atrium.

**Figure 5:**
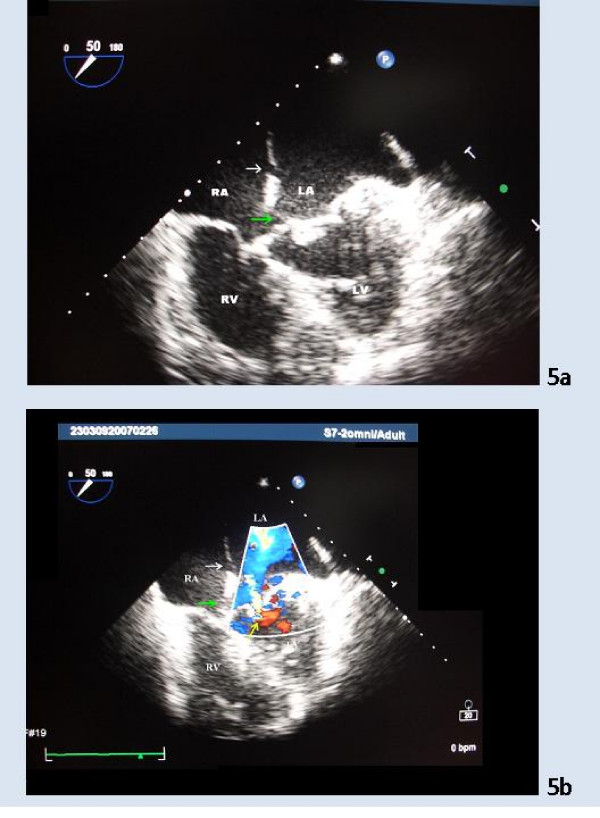
**5a and 5b. TEE images 3 and 4 of the second patient**. The TEE image of the same patient at 50° revealing a small *ostium secundum *atrial defect (white arrow) in addition to the ASD type *ostium primum *(green arrow). Mitral regurgitation is depicted by the yellow arrow (Figure 5b). LA – Left Atrium; RA – Right Atrium; LV – Left Ventricle; RV – Right Ventricle.

Operative treatment was recommended. Patient was put under treatment with Diuretics (due to signs and symptoms of right heart failure), a calcium channel blocker (for Atrial Fibrillation) and Aspirin (for Atrial Fibrillation) until surgical correction would be carried out. Patient refused to take anticoagulant medications with the excuse that she was not able to regularly check the INR.

**Figure 6:**
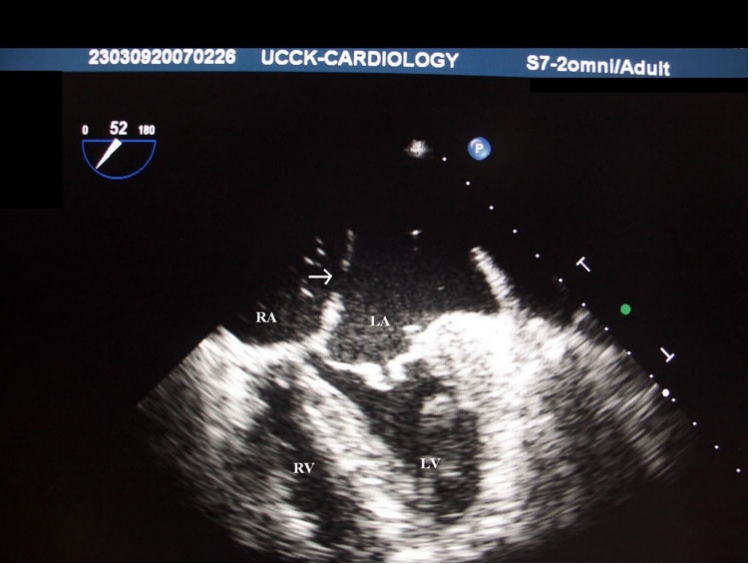
**TEE image 5 of the second patient**. With slight movement of the TEE probe, the ASD *ostium secundum *is visualized more clearly (white arrow). LA – Left Atrium; RA – Right Atrium; LV – Left Ventricle; RV – Right Ventricle.

## Discussion

Both of the cases presented here were diagnosed for the first time at a relatively advanced age. ECG signs of incomplete or complete right bundle branch block are known to be associated with atrial septal defects, however such correlation with other atrial septal abnormalities is not documented. Another ECG sign recognized to be associated with *ostium secundum*-type ASD [7] and PFO [5] is the so called "crochetage", a notched pattern of the R wave in the inferior limb leads. Possible identification of ECG indicators for atrial septal abnormalities would be helpful for early detection of these cardiac disorders, thus preventing on time their numerous consequences.

TEE is a reliable method for examining the atrial septum in adult patients. Hanrath et al. concluded that TEE is superior to transthoracic echocardiography for detection and evaluation of ostium secundum atrial septal defects [8]. TEE was also shown more sensitive than surface echocardiography in detecting PFO [9] and ASA [1]. Mugge et al. reported that ASA, as defined by TEE, was missed by surface echocardiography in almost half (47%) of the patients [1]. In the two cases that we presented, we were able to identify additional atrial septal abnormalities by TEE. In the first case PFO was not seen by surface echocardiography and in the second case ASD type *secundum *was missed.

We defined ASA as a protrusion of the aneurysm of >10 mm beyond the plane of the atrial septum measured by TEE [1], whereas ASA motions were determined according to the classification introduced by Olivares-Reyes et al [2]. ASA combined with PFO are highly associated with cryptogenic stroke and their association has a marked synergistic effect [1,10].

## Conclusion

RBBB in patients with ASA might offer a clue for the presence of other cardiac comorbidities. These cases elucidate, once again, the importance of TEE for accurate diagnosis of atrial septal anomalies.

## Abbreviations

ASA: Atrial Septal Aneurysm; ASD: Atrial Septal Defect; MVP: Mitral Valve Prolapse; PFO: Patent Foramen Ovale; RBBB: Right Bundle Branch Block; TEE: Transesophageal Echocardiography.

## Consent

Written informed consents were obtained from the patients for publication of this case report and accompanying images. Copies of the written consents are available for review by the Editor-in-Chief of this journal.

## Competing interests

The authors declare that they have no competing interests.

## Authors' contributions

AB, LK, EP and AG analyzed and interpreted the patients' data. AB and LK performed transthoracic and TEE procedures. AB was a major contributor in writing the manuscript. All authors read and approved the final manuscript.

## Supplementary Material

Additional file 1 A table has been added to additional files. This table shows some echocardiographic parameters obtained from the two patients. Click here for file
