# Supplementary material for: Atrial septal aneurysm associated with additional cardiovascular comorbidities in two middle age female patients with ECG signs of right bundle branch block: two case reports
Source: Cases J. 2008 Jul 19;1:51. doi: 10.1186/1757-1626-1-51 (PMC2515833; doi:10.1186/1757-1626-1-51)
Supplement: Additional file 1 — A table has been added to additional files. This table shows some echocardiographic parameters obtained from the two patients. [file 1757-1626-1-51-S1.doc]

**Echocardiographic data of the two patients**

|  | **Patient 1** | **Patient 2** |
| --- | --- | --- |
| **Ao (mm)** | 23 | 32 |
| **LA (mm)** | 35 | 42 |
| **RA (mm)** | 39 | 50 |
| **RV (mm)** | 42 | 45 |
| **IVS (mm)** | 9 | 10 |
| **PW (mm)** | 9 | 10 |
| **LVDD (mm)** | 41 | 53 |
| **LVSD (mm)** | 30 | 37 |
| **FS** | 26 | 30 |
| **EF** | 52 | 60 |
| **TR** | 1+ | 3+ |
| **PASP (mmHg)** | 35 | 60 |
| **MR** | / | 2+ |
| **MS** | / | / |
| **AR** | / | 1+ |
| **AS** | / | / |

*Ao: aorta; LA: Left Atrium; RA: Right Atrium; RV: Right Ventricle; IVS: Interventricular Septum; PW: Posterior Wall; LVDD: Left Ventricular Diastolic Dimension; LVSD: Left Ventricular Systolic Dimension; FS: Fractional Shortening; EF: Ejection Fraction; TR: Tricuspid Regurgitation; PASP: Pulmonary Artery Systolic Pressure; MR: Mitral Regurgitation; MS: Mitral Stenosis; AR: Aortic Regurgitation; AS: Aortic Stenosis.
